# Effects of substrate roughness on the fabrication and properties of Ag/AgCl pseudo-reference electrodes for solid-state electrochemical sensors

**DOI:** 10.3389/fchem.2026.1837953

**Published:** 2026-08-21

**Authors:** Ruibang Gao

**Affiliations:** University College London, London, United Kingdom

**Keywords:** chlorination process, electrochemical stability, pseudo-reference electrodes, solid-state electrochemical sensor, substrate roughness

## Abstract

Solid-state silver/silver chloride pseudo-reference electrodes (PREs) are critical for miniaturized, low-cost disposable electrochemical sensors. This work systematically explores the coupling effects of substrate roughness and NaClO chlorination parameters on AgCl film microstructure and electrode electrochemical performance. Magnetron sputtering was used to deposit Cr adhesive layer/Ag functional bilayer films on three substrates with graded surface roughness: baking paper, smooth glass, and mechanically roughened glass. The Ag layers were converted to AgCl via chemical chlorination using sodium hypochlorite solutions with two concentrations (0.5 wt% and 14 wt%). Scanning electron microscopy (SEM) and energy-dispersive X-ray spectroscopy (EDS) were adopted to characterize AgCl nucleation, grain growth, densification, and delamination behaviors, whereas open-circuit potential (OCP) and cyclic voltammetry (CV) measurements quantified electrode potential drift, repeatability, and long-term stability. The results demonstrate that mild 0.5 wt% NaClO combined with optimized chlorination time yields compact, uniform and strongly adherent AgCl coatings on all substrates; the optimal durations are 240 s for baking paper, 210 s for roughened glass and 90 s for smooth glass. In contrast, 14 wt% high-concentration NaClO accelerates initial nucleation yet induces severe grain coarsening and structural defects. Surface roughening effectively enhances interfacial anchoring of AgCl layers and alleviates structural degradation under extended chlorination. This comparative study provides straightforward guidance for fabricating high-stability coating-free solid-state PREs.

## 1 Introduction

As important devices that respond to external chemical or physical stimuli, sensors have evolved from early large-scale instruments into highly portable, low-power-consumption, and intelligent microdevices to meet the demand for rapid information acquisition in various scenarios (Baranwal et al., 2022; Singh et al., 2024). Among numerous sensing technologies, electrochemical sensors have attracted considerable attention owing to their ability to directly convert chemical signals into electrical signals. Compared with optical and thermal detection methods, electrochemical sensors exhibit advantages including simple structure, easy integration, low cost, and facile regulation and acquisition of output signals (voltage and current). Consequently, they have been widely applied in fields such as medical diagnosis, environmental monitoring, and food safety (Khan et al., 2024; Yeung et al., 2021; Zhao et al., 2025).

In a conventional three-electrode electrochemical system, which is standard for transient electrochemical methods, the reference electrode provides a stable potential reference for the working electrode (WE), and the counter electrode acts as the third electrode to close the electrical circuit, thus ensuring the accuracy and repeatability of measurements (Anantharaj et al., 2023; Troudt et al., 2022). An ideal reference electrode should exhibit non-polarizable behavior, with its potential conforming to the Nernst equation and maintaining high stability. However, traditional liquid reference electrodes rely on internally filled liquid electrolytes and complex porous diaphragm structures (Kasem and Jones, 2008), which are plagued by several limitations including large volume, high susceptibility to electrolyte leakage or contamination, impeded system miniaturization and integration, as well as poor stability under extreme conditions such as high temperature and high pressure. Although gel electrolyte systems can replace liquid electrolytes to a certain extent, they still face issues like gel aging and water volatilization during long-term service.

The emergence of solid-state reference electrodes provides an effective solution to overcome the constraints (Gao et al., 2020; Subbiah et al., 2025; Yang and Suzuki, 2024). By substituting the internal liquid junction system with a solid-state membrane, these electrodes significantly facilitate the miniaturization of electrode structures and fundamentally mitigate the risk of leakage. Among them, reference electrodes based on the Ag/AgCl system have garnered widespread attention due to their non-toxicity, stable chemical properties, and relatively simple preparation process (Dawkins et al., 2021; Wang et al., 2024; Liang et al., 2025). Their stable potential originates from the thermodynamic equilibrium established by the reversible redox reaction of AgCl/Cl^−^.

Furthermore, Ag/AgCl pseudo-reference electrodes, which eliminate the internal KCl electrolyte chamber, feature a more simplified structure and lower cost, making them an ideal choice for disposable and portable sensing systems. Nevertheless, the potential of such electrodes is directly affected by the chloride ion activity in the external test solution, and the structural stability of the solid-state AgCl membrane is crucial. As a result, their long-term potential stability is generally inferior to that of traditional liquid reference electrodes. Therefore, optimizing the microstructure of the AgCl membrane through process regulation is the key to enhancing its performance.

At present, research on improving the stability of solid-state Ag/AgCl reference electrodes mainly focuses on post-modification of the membrane layer, such as coating KCl-doped gel layers or polymer protective films. However, relatively few studies have been conducted on the key preparation parameters that determine the initial structure of the AgCl membrane, including factors such as chlorinating agent concentration, chlorination reaction time, and substrate surface roughness. Therefore, investigating the influence laws of substrate properties and chlorination process parameters on the formation process and electrochemical stability of the AgCl membrane is of great significance for providing experimental basis and theoretical reference for the development of high-stability, low-cost, and flexibly integrable solid-state pseudo-reference electrodes.

## 2 Related works

Solid-state reference electrodes have been extensively investigated over the past 2 decades. Their core scientific issue lies in constructing a solid-state interface capable of maintaining a stable Ag/AgCl/Cl^−^ equilibrium without relying on internal liquid electrolytes, thereby providing a reliable and reproducible reference potential. Early studies mainly employed gel electrolytes or polymer electrolyte membranes as Cl^−^ sources and ionic conductors to mitigate potential drift (Michalska, 2012; Yoon et al., 2000). Although such methods can effectively improve stability in the short term, issues including water evaporation in the membrane, uneven ionic migration rates, and insufficient mechanical strength render their long-term performance unable to meet the stringent requirements of disposable sensors or on-site rapid detection.

With the advancement of micro/nanofabrication technology, the direct fabrication of Ag/AgCl PRE without internal electrolytes via thin-film technology has emerged as a new research mainstream (Nehal et al., 2020; Xu et al., 2021). Such electrodes generate an AgCl layer through *in-situ* chlorination of the deposited silver film, thereby directly establishing a potential equilibrium with the external solution. Chlorination reactions are typically achieved using oxidizing agents such as FeCl_3_, HCl, KCl or NaClO. Among these, sodium hypochlorite (NaClO) has been widely adopted for the chlorination of thin silver films due to its fast reaction rate, simple operation, and facile precise control (Mirzaei et al., 2025; Su et al., 2022). The performance of the AgCl film—including nucleation density, particle size, porosity, and adhesion to the substrate—is the key factor determining the electrochemical stability of PRE. These structural characteristics are synergistically influenced by multiple process parameters such as chlorinating agent concentration, reaction time, substrate surface properties, and initial silver film thickness.

At the level of preparation processes, silver films can be obtained via methods such as screen printing, thermal evaporation, and magnetron sputtering. Among these methods, magnetron sputtering technology has been widely adopted due to its ability to fabricate silver films with high density, precisely controllable thickness, and good adaptability to various irregular substrates (Iconaru et al., 2022; Rashid et al., 2021; Zhang et al., 2021). However, during the chlorination process, the conversion of silver films to AgCl is accompanied by significant volume expansion, which tends to induce issues including internal stress accumulation, abnormal grain growth, and even film delamination. These problems impair the uniformity and continuity of the resultant films, thereby leading to potential drift. In particular, the localized formation of coarse AgCl grains may trigger film cracking or complete detachment, which severely undermines the stability of PRE (Lim et al., 2020).

The influence of substrate material properties on the structure of AgCl films has also attracted increasing attention (Cho et al., 2022; Dawkins et al., 2021; Xu et al., 2024). The surface hydrophobicity and roughness morphology of substrates significantly affect the nucleation and growth behaviors of AgCl. Hydrophobic surfaces tend to result in sparse nucleation sites, while hydrophilic surfaces or those subjected to roughening treatment are conducive to increasing nucleation density and enhancing film adhesion. The micro-fibrous or rough structures on the surface of flexible substrates can provide more mechanical anchoring sites for AgCl films, thereby improving their stability under prolonged chlorination or mechanical bending (Sun et al., 2020; Sun et al., 2025). However, excessive surface roughness may also lead to discontinuities in the initially deposited silver films, which in turn exerts an adverse effect on the potential stability of the final electrodes. Therefore, the synergistic optimization of substrate characteristics and chlorination process parameters is of great importance.

The potential analysis and drift mechanism of PRE represent another research focus (Cengiz et al., 2021; Papamatthaiou et al., 2020; Ruengpirasiri et al., 2023). Due to the absence of internally fixed Cl^−^ activity, the potential of PRE is extremely sensitive to the conductivity, ionic strength, pH value, and interfacial adsorption behavior of the external solution. Via analytical techniques including open-circuit potential, cyclic voltammetry, and electrochemical impedance spectroscopy, researchers have confirmed that AgCl films with high compactness, good continuity, and uniformity can effectively reduce potential drift and improve the potential reproducibility of electrodes in different solution systems.

Although existing studies have achieved considerable progress in the material systems, preparation processes, and membrane modification of solid-state reference electrodes, systematic research on the correlations and comparisons among substrate properties, chlorination kinetics, the microstructure of the obtained AgCl films, and their ultimate electrochemical stability remains insufficient. There is a lack of differential analysis on the structural and performance differences of AgCl films formed on substrates of different materials under the same chlorination conditions, as well as the clarification of the mechanism by which such structural differences quantitatively affect the stability of PRE. Therefore, this study aims to investigate the synergistic influence laws of substrate roughness and chlorination parameters on the formation process and electrochemical performance of AgCl films, thereby providing a theoretical basis for the rational design and controllable fabrication of high-performance solid-state PRE.

## 3 Materials and methods

### 3.1 Material substrate

Three substrate materials with gradient surface roughness, namely, baking paper, smooth glass, and roughened glass, were selected to systematically reveal the coupling effects of substrate physicochemical properties on thin-film deposition, chlorination kinetics, and the final performance of Ag/AgCl PRE. All substrates underwent unified and standardized pre-chlorination pretreatment to ensure consistent initial surface cleanliness and eliminate exogenous contaminants before silver sputtering and chlorination experiments.

Standard commercial baking paper was used without additional surface modification or chemical treatment. Universal commercial baking paper with uniform cellulose fiber structure is applicable for this study, with no strict restrictions on manufacturers or minor roughness deviations. Before use, the baking paper was placed in a dry and dust-free environment for 24 h to remove surface adsorbed moisture and fine dust particles. Initial surface characterization confirms that baking paper possesses inherent microscale porous and rough fibrous structures, with stable surface chemical composition dominated by cellulose, without residual reactive groups.

Smooth glass is a chemically inert amorphous silica substrate with a relatively uniform and homogeneous surface. Strict pre-treatment was implemented: the glass was ultrasonically cleaned in acetone, ethanol, and 18.2 MΩ cm DI water sequentially for 900 s each, followed by nitrogen blow-drying. Based on the substrate preparation method and the observed SEM morphology, the roughened glass substrate is expected to possess a higher surface roughness than the smooth glass substrate.

The roughened glass substrate was prepared via a standard mechanical wet polishing method: original float glass was sequentially polished with 800-mesh, 1200-mesh, and 2000-mesh silicon carbide abrasive papers under continuous DI water flushing to avoid residual scratches, particle contamination, and thermal surface damage. This layered wet polishing can strip the outermost glass layer carrying inherent metallic impurities from float-glass manufacturing. After polishing, the roughened glass adopted the same three-stage ultrasonic cleaning and nitrogen drying procedure as smooth glass to further eliminate residual contaminants and ensure consistent surface cleanliness before experiments.

In addition to baking paper, other cellulose-based papers (filter paper, chromatographic paper) have similar porosity and roughness, but baking paper was selected for its uniform fiber distribution, excellent oil resistance, and high compatibility with disposable electrochemical sensor fabrication. Although glass substrates can be artificially etched or textured to obtain higher porosity and roughness, they cannot replicate the flexible three-dimensional fibrous network and stress-buffering effect of baking paper. The characteristics and selection criteria of the three substrates are presented in Table 1.

**TABLE 1:** Characteristics and selection criteria of three types of material substrates.

Substrate type	Material composition and structure	Surface roughness	Selection rationale
Baking paper	Cellulose network with natural porosity and flexibility	High (microscale fibrous roughness)	Flexible and low-cost
Smooth glass	Inorganic amorphous silica with dense and chemically homogeneous surface	Low	Rigid and smooth
Roughened glass	Same as smooth glass, treated with abrasives	Medium	Control group

### 3.2 Preparation method and process

A two-step method combining magnetron sputtering and solution-based chemical chlorination was adopted to fabricate Ag/AgCl PRE (Su et al., 2022; Xu et al., 2021). The advantage of this method lies in that the sputtering process enables precise control over the thickness and uniformity of the metal film, while solution chlorination allows flexible modulation of the morphology and structure of the AgCl layer. The design of the entire process aims to systematically elucidate the influence mechanism of key variables such as substrate type, chlorinating agent concentration, and reaction time on the ultimate performance of the electrodes.

#### 3.2.1 Sputter deposition of Cr/Ag bilayer films

Firstly, magnetron sputtering technology was employed to sequentially deposit a chromium (Cr) adhesion layer and a silver (Ag) functional layer on all pretreated substrates (baking paper, smooth glass, and roughened glass). The sputtering chamber was pumped to a base vacuum below 3 × 10^−4^ Pa before deposition; high-purity argon was filled as sputtering gas and maintained at a constant working pressure of 0.6 Pa throughout the deposition, with DC power set at 80 W for Cr target and 120 W for Ag target, and target-to-substrate distance fixed at 90 mm. Prior to Ag layer deposition, a 10 nm-thick Cr layer was sputtered onto the substrates. Chromium exhibits excellent chemical affinity with most substrates, enabling the formation of strong chemical bonds; meanwhile, it also possesses good compatibility with the overlying Ag film. This adhesion layer can effectively alleviate the interfacial stress caused by thermal expansion coefficient mismatch, thereby preventing the subsequent Ag film and AgCl layer from peeling off the substrates. Subsequently, the deposition of the Ag functional layer was carried out. A 200 nm-thick Ag film was further sputtered onto the Cr layer. This thickness was optimized to ensure the formation of a continuous, dense conductive layer with low sheet resistance, while simultaneously providing a sufficient material volume for subsequent chlorination to generate an AgCl layer with a specific thickness. The entire sputtering process was conducted at room temperature to avoid substrate deformation or excessive internal stress in the films induced by thermal effects.

#### 3.2.2 Chemical chlorination for fabrication of AgCl active layer

The samples deposited with Ag films were converted into AgCl active layers via chemical chlorination. In this process, aqueous sodium hypochlorite (NaClO) solution was selected as the chlorinating agent, and the core reaction involved is a redox process. The ClO− ions in the solution oxidize metallic Ag to form solid AgCl adhering to the surface. NaClO was chosen because of its fast reaction rate, simple by-products, and facile precise control over reaction conditions.

To systematically investigate the effects of chlorination kinetics on the morphology and structure of AgCl films, two key variables were designed in the experiment: chlorinating agent concentration and reaction time. In terms of concentration, two significantly different gradients were selected, namely, 0.5 wt% (low concentration) and 14 wt% (high concentration). Precise time intervals were strictly controlled for all chlorination experiments: for baking paper with 0.5 wt% NaClO, time intervals were 30 s, 60 s, 120 s, 180 s, 210 s, 300 s; for smooth/roughened glass with 0.5 wt% NaClO, time intervals were 30 s, 60 s, 90 s, 120 s, 150 s, 180 s; for baking paper with 14 wt% NaClO, time intervals were 20 s, 40 s, 60 s, 80 s, 100 s. All time points were chosen at equal intervals to ensure accurate tracking of AgCl nucleation–growth–densification–peeling evolution. The optimal time for each substrate was determined as the point where AgCl film became fully continuous, uniform, and dense without cracks or delamination, which was verified by SEM, EDS, OCP, and CV collectively.

In the time dimension, different continuous time gradients were set for each concentration. This enables dynamic capture and clarification of the complete formation and evolution mechanism of the AgCl layer: starting from the initial heterogeneous nucleation, followed by the lateral growth of grains to cover the entire surface, then vertical thickening, and finally film cracking or peeling off the substrate due to volume expansion stress in the case of excessive growth. Through this multi-variable coupled experimental design, the intrinsic correlation between chlorination process parameters and film microstructure can be established.

During the chlorination operation, each prepared sample was cut into 10 mm × 15 mm specimens, and every single specimen was immersed vertically into 10 mL freshly prepared NaClO aqueous solution of specified concentration, ensuring that the liquid level completely covered the reaction area. A fixed liquid-to-sample ratio was strictly kept for all chlorination batches to eliminate volume-induced reaction deviation. After reaching the present time, the sample was promptly taken out and immediately rinsed under flowing deionized water for at least 30 s to thoroughly terminate the reaction and remove residual oxidizing ions. Finally, the sample was gently purged with a stream of clean, dry nitrogen along the surface, and stored in a desiccator after removing moisture for subsequent characterization and analysis.

### 3.3 Structural and compositional characterization and analysis methods

SEM coupled with EDS was used to characterize AgCl microstructure and elemental composition, linking fabrication parameters to film chemical and morphological features (Dong et al., 2021; Wu et al., 2023; Yoon et al., 2021).

#### 3.3.1 Morphology and structure analysis

Field-emission SEM was used to characterize surface morphology. To obtain a high-quality conductive surface, all samples were subjected to gold sputtering treatment prior to characterization. Systematic observations were conducted at different magnifications, with a focus on analyzing the following three interrelated structural dimensions.

Firstly, the nucleation and growth behaviors of AgCl were observed, including the initial nucleation density, grain distribution uniformity, and the evolution of growth modes with prolonged chlorination time. Secondly, the macroscopic quality of the film was evaluated, such as its continuity, compactness, surface coverage, as well as the presence of defects including microcracks, voids, or local delamination. Finally, the differential effects of different chlorination process parameters on the final morphology were systematically compared. Under the high-concentration NaClO condition, investigations were focused on whether excessive reaction driving force would induce abnormal grain coarsening, loose stacking, or cracking caused by volume expansion stress. These microstructural characteristics are the key basis directly correlated with the macroscopic electrochemical stability of the electrodes.

#### 3.3.2 Chemical composition and distribution analysis

SEM-equipped EDS was applied to map and quantify Ag and Cl elemental signals. Point composition measurements were performed on representative micro-regions, such as specific grains, crack edges, or suspected unreacted areas, to obtain the accurate Cl/Ag atomic ratio, thereby directly evaluating the local chlorination degree. Meanwhile, elemental mapping images of Ag and Cl were acquired for selected regions to intuitively visualize the distribution uniformity of Cl in the films and identify the presence of incompletely chlorinated areas caused by diffusion or reaction inhomogeneity.

By systematically comparing the EDS results of different samples, this study aimed to confirm the reaction completeness and uniformity from a chemical perspective, which mutually corroborated with the SEM morphological observations to jointly reveal the key factors affecting the quality of AgCl films.

### 3.4 Electrochemical performance analysis methods

All electrochemical characterizations were carried out in a standard three-electrode cell to assess the potential stability of as-fabricated PREs (Ball, 2022; Macedo et al., 2024; Torres-Gonzalez et al., 2022). OCP tests tracked time-dependent potential drift, a key metric for PRE stability. Potential drift is the combined result of multiple factors, including membrane structural integrity, interfacial Cl^−^ exchange kinetics, double-layer stability, and internal stress relaxation. Theoretically, an AgCl film with fine grains, continuous and dense morphology, and strong adhesion can provide a more stable and reversible Ag/AgCl/Cl^−^ interface, thereby exhibiting lower noise levels and a slower drift rate. Conversely, if the film contains coarse grains, cracks, or local delamination, it may lead to significant potential fluctuations, jumps, or even continuous drift.

CV with ferricyanide/ferrocyanide redox probe further validated PRE potential stability via tracking redox peak shifts across scanning cycles. If the PRE potential exhibits drift or fluctuation, it will manifest as systematic shifts in redox peak positions or peak shape distortion in different scanning cycles. Combining the long-term stability information provided by OCP measurements with the transient perturbation responses revealed by CV tests enables a comprehensive and in-depth elucidation of the influence mechanisms of different substrate characteristics and chlorination process parameters on the electrochemical performance of PRE, thereby establishing a complete correlation framework from microstructure to macroscopic performance.

All OCP and CV electrochemical measurements were carried out in 3.5 Mol/L KCl aqueous electrolyte. For CV characterization, 1 mM equimolar mixture of K_3_ [Fe(CN)_6_]/K_4_ [Fe(CN)_6_] was added as redox probe into the above KCl supporting electrolyte. Meanwhile, all electrochemical measurements were performed on at least three independently fabricated electrodes for each combination of substrate and chlorination condition. The representative OCP and CV curves shown in the figures were selected from these replicate measurements.

## 4 Experiment and analysis

Three substrates with gradient surface roughness were tested to decouple material and roughness effects on PRE performance. Roughened glass, with the same chemical composition as smooth glass but a different surface morphology due to physical polishing, served as a control to isolate the effect of surface roughness on AgCl adhesion and electrode stability. Electrochemical evaluations including OCP drift and CV repeatability were used to correlate stability differences across the three substrate types.

### 4.1 Materials and instruments

The main materials used in the experiment were as follows: glass substrates (RS France, Model No. BPB018), roughened glass substrates, and baking paper. The main reagents and consumables employed were listed as follows: silver target (99.99% purity, Testbourne Ltd., Model No. S5-9000-D118), chromium target (99.99% purity, Testbourne Ltd., Model No. S5-9000-D118), sodium hypochlorite solution (NaClO, available chlorine content 14%, VWR Chemicals), isopropanol (IPA), potassium chloride (KCl, purity > 99.0%, Alfa Aesar), potassium hexacyanoferrate (II) trihydrate (K_4_ [Fe(CN)_6_]·3H_2_O, Vital Minerals), potassium hexacyanoferrate (III) (K_3_ [Fe(CN)_6_], Merck Group, Sigma-Aldrich), commercial silver reference electrode (Metrohm DropSens, Model No. DRP-110-U75), deionized water, and Ag/AgCl (KCl solution) reference electrode (BASi Research Products, Model No. MF-2056).

**FIGURE 1:**
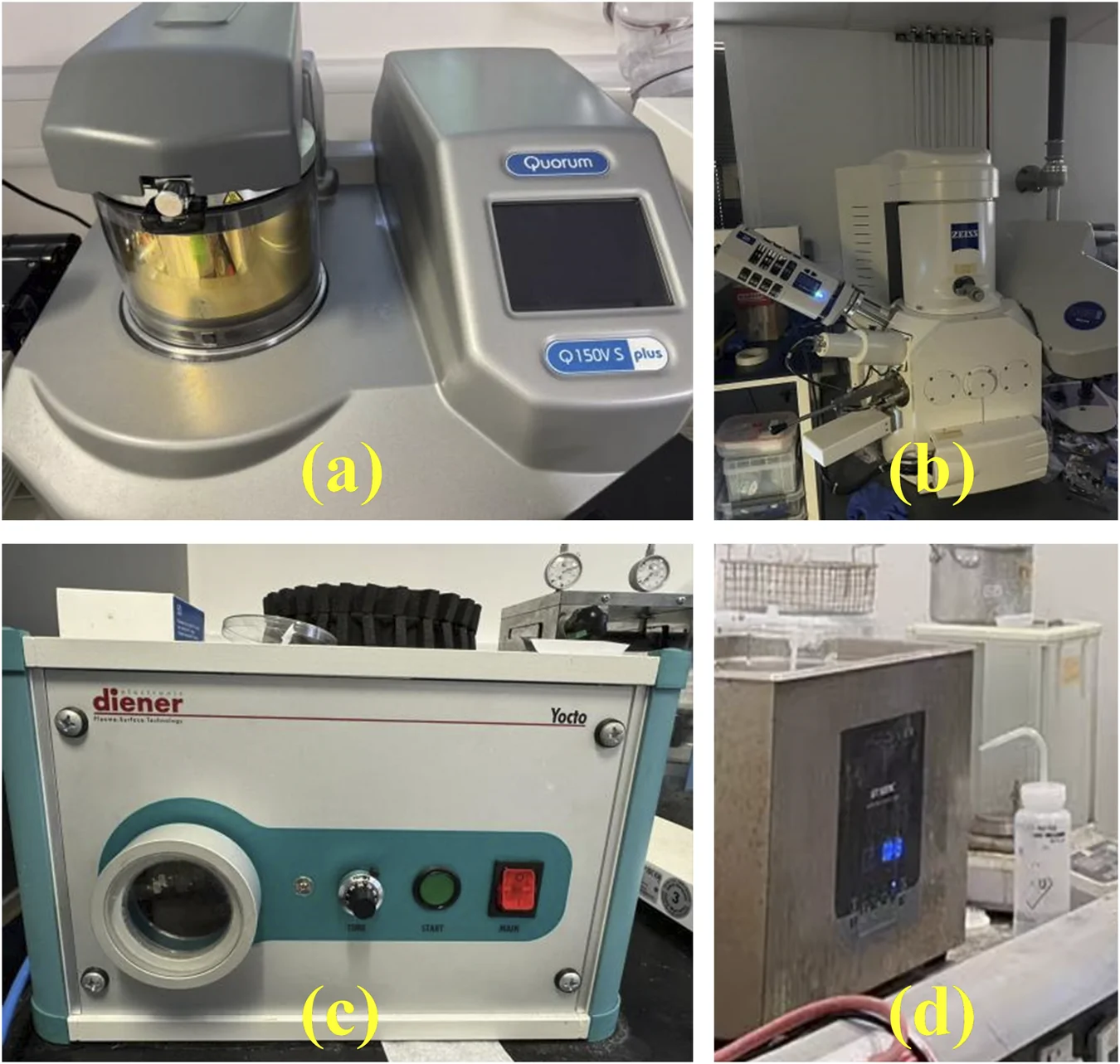
Experimental instruments for **(a)** Magnetron sputtering apparatus, **(b)** EDX and SEM, **(c)** Plasma cleaner and **(d)** Ultrasonic Cleaner.

The instruments and equipment used in this study included the following: the Q150V S Plus ultra-high vacuum sputtering coating system, Diener Electronic Yocto III plasma treatment system, and GT SONIC-D20 ultrasonic cleaner for sample preparation and processing; the scanning electron microscope (SEM) manufactured by Carl Zeiss AG, Germany, coupled with its ZEISS Smart EDX energy dispersive X-ray spectroscopy system for morphology observation and composition analysis; and the electrochemical workstation for all electrochemical performance tests, as shown in Figure 1.

All Deionized water used in the experiments had a resistivity of 18.2 MΩ cm (Milli-Q integral system). Electrochemical measurements were performed using a Metrohm Autolab PGSTAT128N potentiostat. For CV tests, the scan speed was set to 50 mV/s in a potential window from −0.6 V to 0.8 V. SEM characterization was carried out at an acceleration voltage of 10.00 kV with a probe current of 100 pA. All experiments were conducted at room temperature (25 °C ± 1 °C).

### 4.2 SEM analysis

Pre-chlorination Ag film morphology was first characterized to eliminate native substrate defects as confounding variables. All commercial substrates inherently contain intrinsic material defects such as crystal defects or fibrous voids, while the sputtered silver coatings deposited on all three substrates are uniform, intact, and free from fabrication-induced defects including pinholes, cracks, and delamination before chlorination. These native substrate defects will not affect the subsequent comparative analysis of AgCl evolution, as the initial Ag layer maintains consistent deposition quality across all samples. Specifically, the sputtered Ag film fully and uniformly covered the natural cellulose network of baking paper without voids or peeling. The smooth glass substrate carried a dense, continuous planar Ag layer with no structural imperfections on its ultra-flat surface. For roughened glass, the deposited Ag film closely conformed to the polished microscale concave–convex textures, achieving homogeneous coverage without local fracture or uneven deposition. This consistent and high-quality initial film state confirms that all subsequent morphological differences after chlorination originate exclusively from substrate surface properties and chlorination conditions, rather than pre-existing fabrication defects.

**FIGURE 2:**
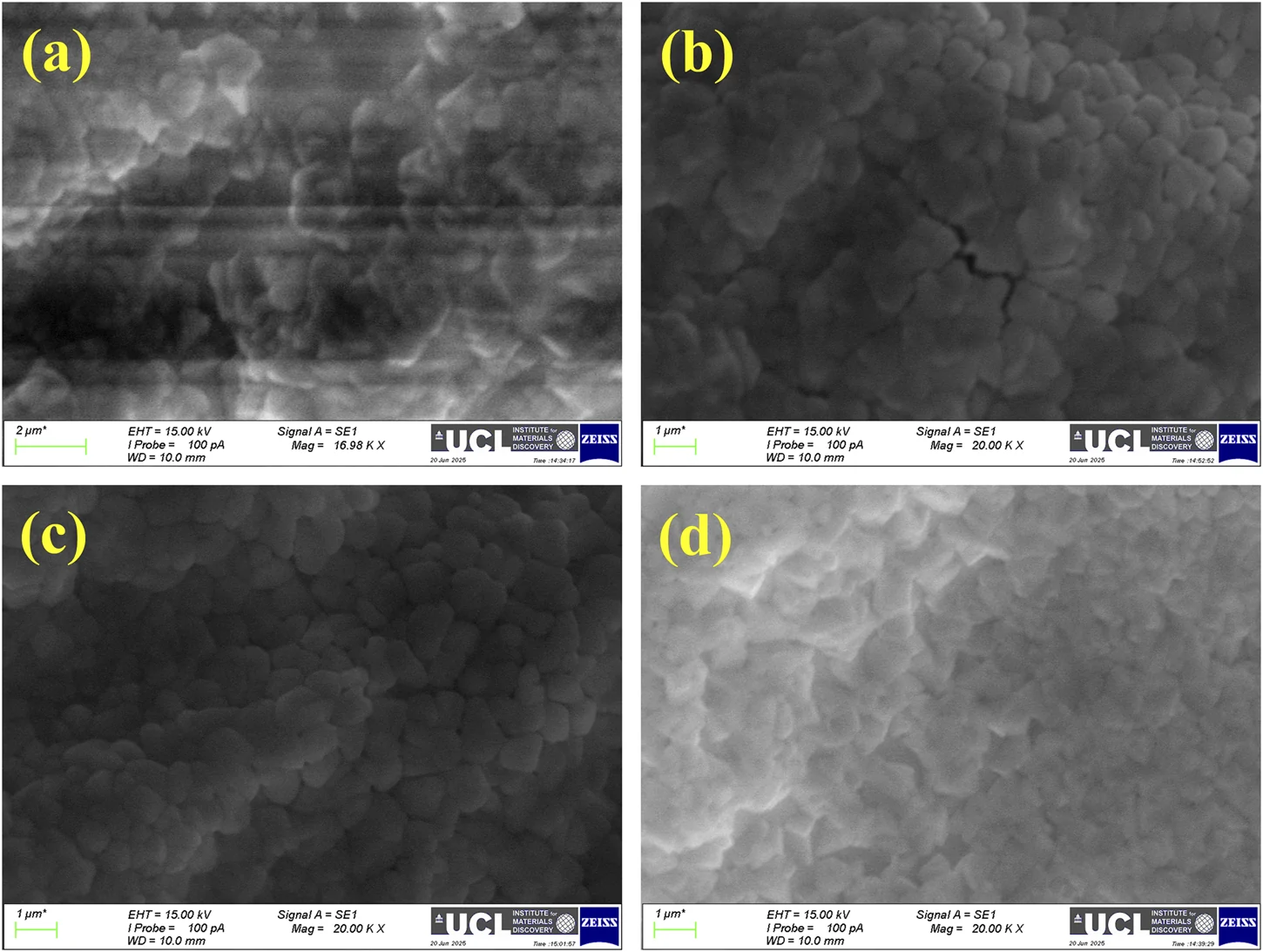
Representative SEM images of AgCl morphology on baking paper substrate after 0.5 wt% NaClO chlorination for **(a)** 120 s, **(b)** 180 s, **(c)** 240 s, and **(d)** 300 s. Images are representative of observations from at least three regions per sample. Representative data are from multiple independent measurements.

Under the same baseline, baking paper showed the most favorable AgCl evolution due to its flexible, micro-rough fibrous structure as shown in Figure 2. The cellulose network appears to provide abundant nucleation sites, inferred from the denser and more uniform AgCl particle distribution in SEM images. The fibrous structure may also accommodate volume changes during the Ag-to-AgCl transition, as suggested by the absence of extensive cracking compared to smooth glass, consistent with a potential mechanical interlocking effect that enhances film adhesion.

**FIGURE 3:**
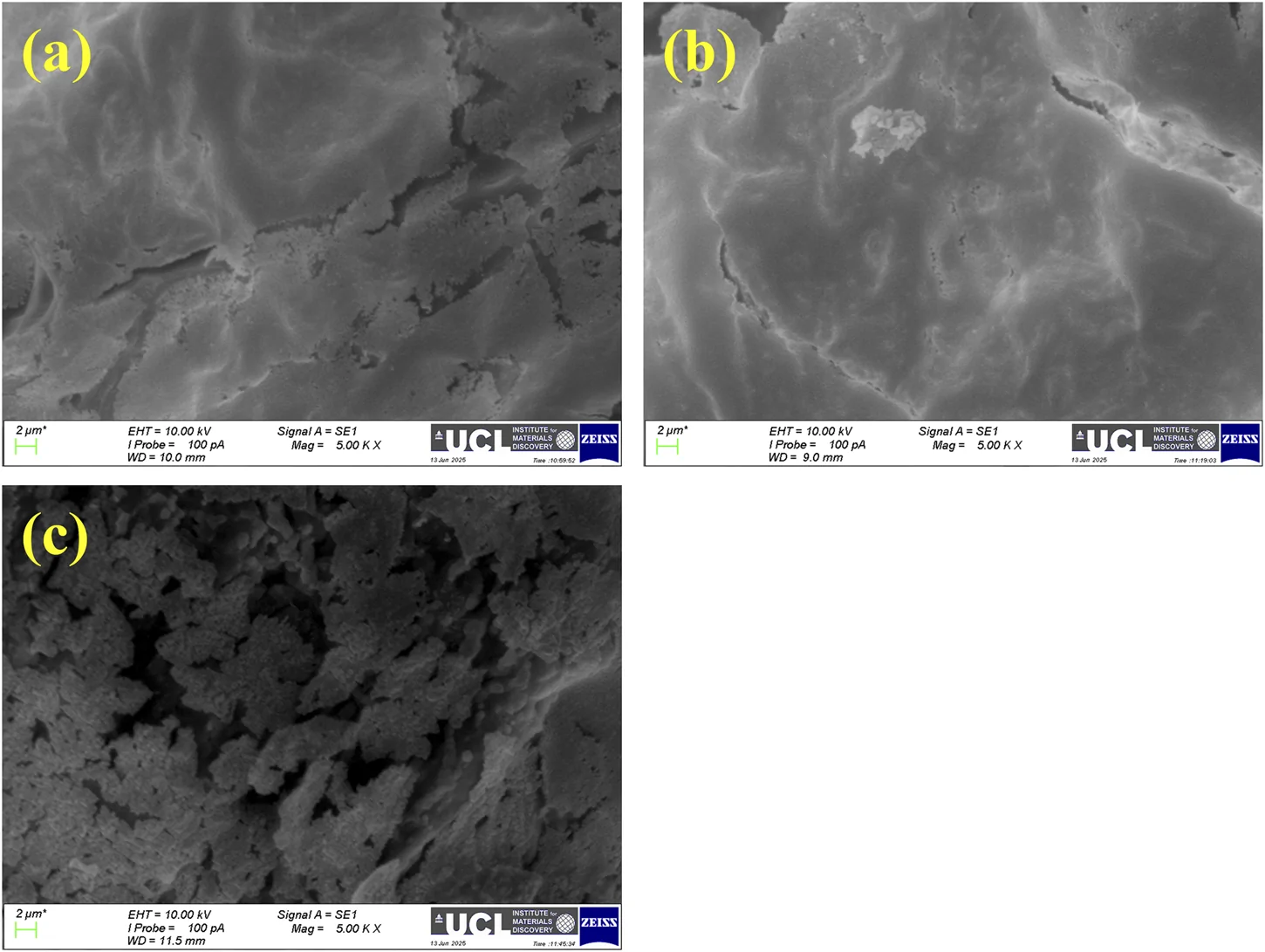
Representative SEM images of AgCl morphology on baking paper substrate after 14 wt% NaClO chlorination for **(a)** 60 s, **(b)** 80 s, and **(c)** 100 s. Representative data are from multiple independent measurements.

In sharp contrast, high-concentration 14 wt% NaClO triggers uncontrollable and destructive chlorination kinetics on baking paper as shown in Figure 3. The strong oxidizing and high-Cl^-^ environment drastically accelerates the phase transformation rate, leading to rapid and excessive volume expansion of the AgCl film. As a typical symptom of over-chlorination, distinct structural cracks appear at around 100 s, accompanied by severe grain coarsening and structural loosening. Such irreversible structural failure demonstrates that high-concentration chlorination induces unrelievable interfacial stress, destroys film compactness, and inevitably causes electrochemical performance deterioration.

**FIGURE 4:**
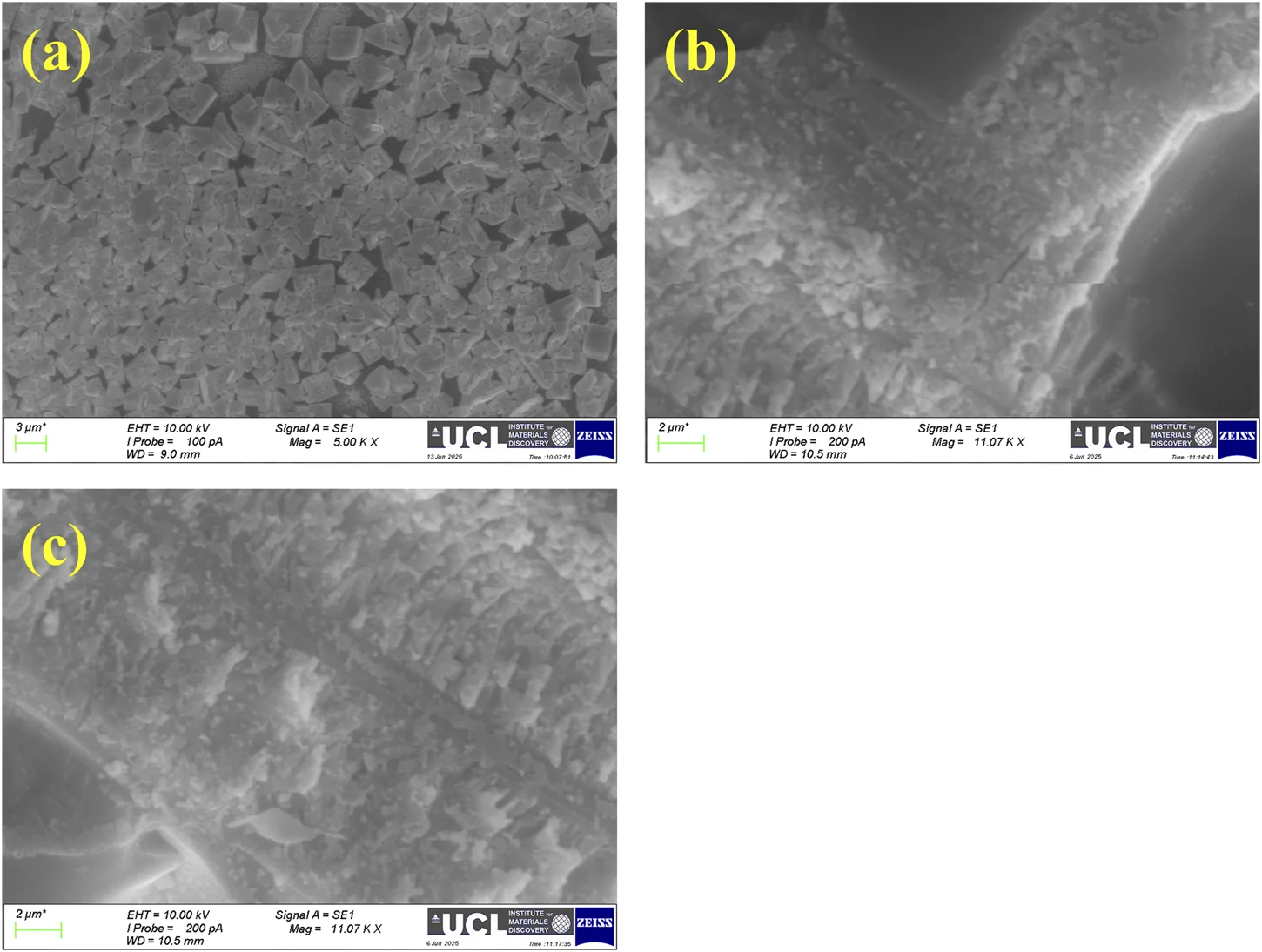
Representative SEM images of AgCl morphology on smooth glass substrate after 0.5 wt% NaClO chlorination for **(a)** 90 s, **(b)** 120 s, and **(c)** 150 s. Representative data are from multiple independent measurements.

In contrast, although the smooth glass substrate allows for the deposition of a dense and continuous initial Ag film, its ultra-smooth and rigid surface with negligible micro-undulation lacks microscopic anchoring sites and stress-buffering capacity. This ultra-flat characteristic severely facilitates the accumulation of unrelieved volume expansion stress at the interface during the subsequent chlorination process, which originates from the massive phase transformation of Ag to AgCl. As illustrated in Figure 4, under the mild condition of 0.5 wt% NaClO solution, the glass substrate exhibits a relatively narrow optimal chlorination window, and a uniformly covered AgCl layer can be obtained at a reaction time of approximately 90 s. Nevertheless, further prolongation of the chlorination time leads to the generation of microcracks and local delamination in the AgCl layer, because the stress cannot be effectively released via substrate deformation or mechanical interlocking. This phenomenon cannot be merely attributed to low interfacial adhesion. The observed differences in crack and delamination behavior across substrates suggest that substrate flexibility may play an important role in stress relief during the volume expansion of AgCl. Rigid and smooth glass substrates lacking stress-buffering capability are more susceptible to microcrack formation and film delamination. Notably, when treated with a high-concentration 14 wt% NaClO solution, the AgCl film grown on the smooth glass substrate suffers from rapid large-area delamination caused by stress concentration, failing to form a stable structure that meets the requirements for reliable electrochemical measurements. Therefore, the investigation of the effect of high-concentration chlorinating agents on electrode performance was only carried out on baking paper substrates with excellent interfacial adhesion, to guarantee the validity and comparability of the experimental data.

**FIGURE 5:**
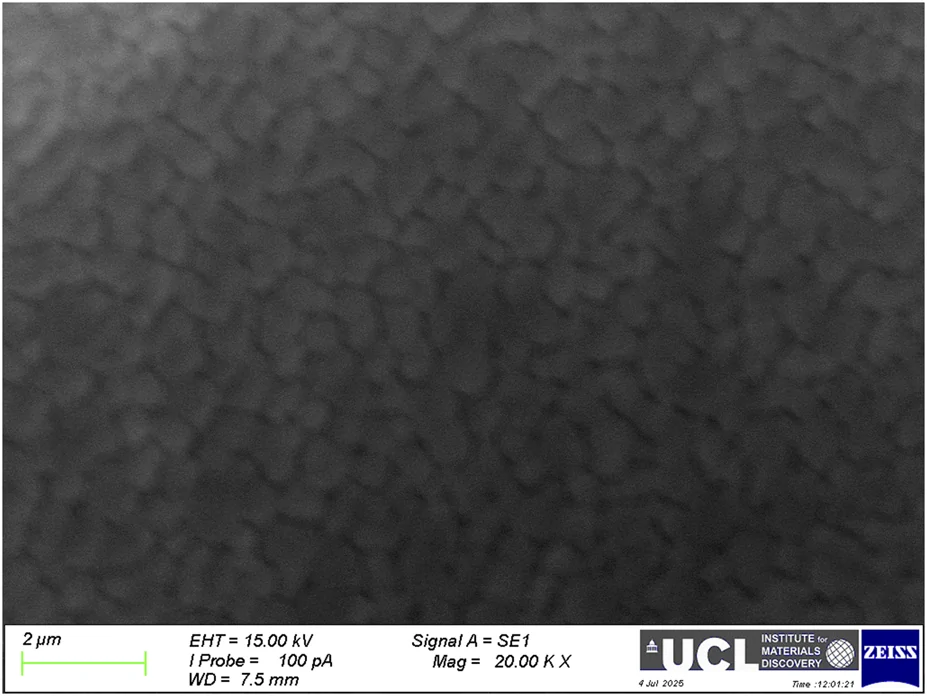
Representative SEM image of AgCl morphology on roughened glass substrate after 210 s of 0.5 wt% NaClO chlorination. Representative data are from multiple independent measurements.

The chlorination behavior and structural characteristics of the roughened glass substrate fall between those of the baking paper and smooth glass substrates, further confirming the critical role of substrate surface morphology. The micron-scale grooves and protrusions formed on its surface via physical polishing constitute reproducible physical roughness. This structure provides more nucleation sites than the smooth glass substrate during the deposition stage, thereby significantly improving the distribution uniformity of AgCl particles after chlorination, as shown in Figure 5. More importantly, the rough surface increases the effective reactive contact area between the Ag film and the chlorination solution, and creates additional mechanical anchoring sites at the interface. This enables AgCl to form a continuous thin film more rapidly in the early stage of the chlorination reaction. For roughened glass substrates, chlorination experiments using 14 wt% NaClO were also not performed. Although roughened glass provides better adhesion than smooth glass, the extremely high oxidation strength of 14 wt% NaClO still induces rapid and excessive AgCl grain coarsening, severe volume expansion stress, and early film delamination. This would result in structurally invalid electrodes with irreproducible performance, making such experiments scientifically unnecessary and unable to provide valid comparative data. Therefore, high-concentration chlorination tests were limited to baking paper, which has the strongest film adhesion and structural tolerance.

Only one representative time point is presented for roughened glass because this time corresponds to the optimized, stable, and structurally ideal AgCl layer used in all electrochemical tests. Shorter times lead to incomplete chlorination, while longer times cause minor grain coarsening but do not change the key conclusion that roughness improves adhesion. Since the formation trend of AgCl on roughened glass is highly similar to on smooth glass, a full time series is redundant and does not provide additional mechanistic insight. The single optimized time point is sufficient to support the core conclusion about the effect of substrate roughness. SEM analysis reveals the morphological evolution of AgCl films on the three types of substrates, indicating that substrate roughness is one of the key factors influencing the uniformity, adhesion, and structural stability of AgCl films.

### 4.3 EDS analysis

**FIGURE 6:**
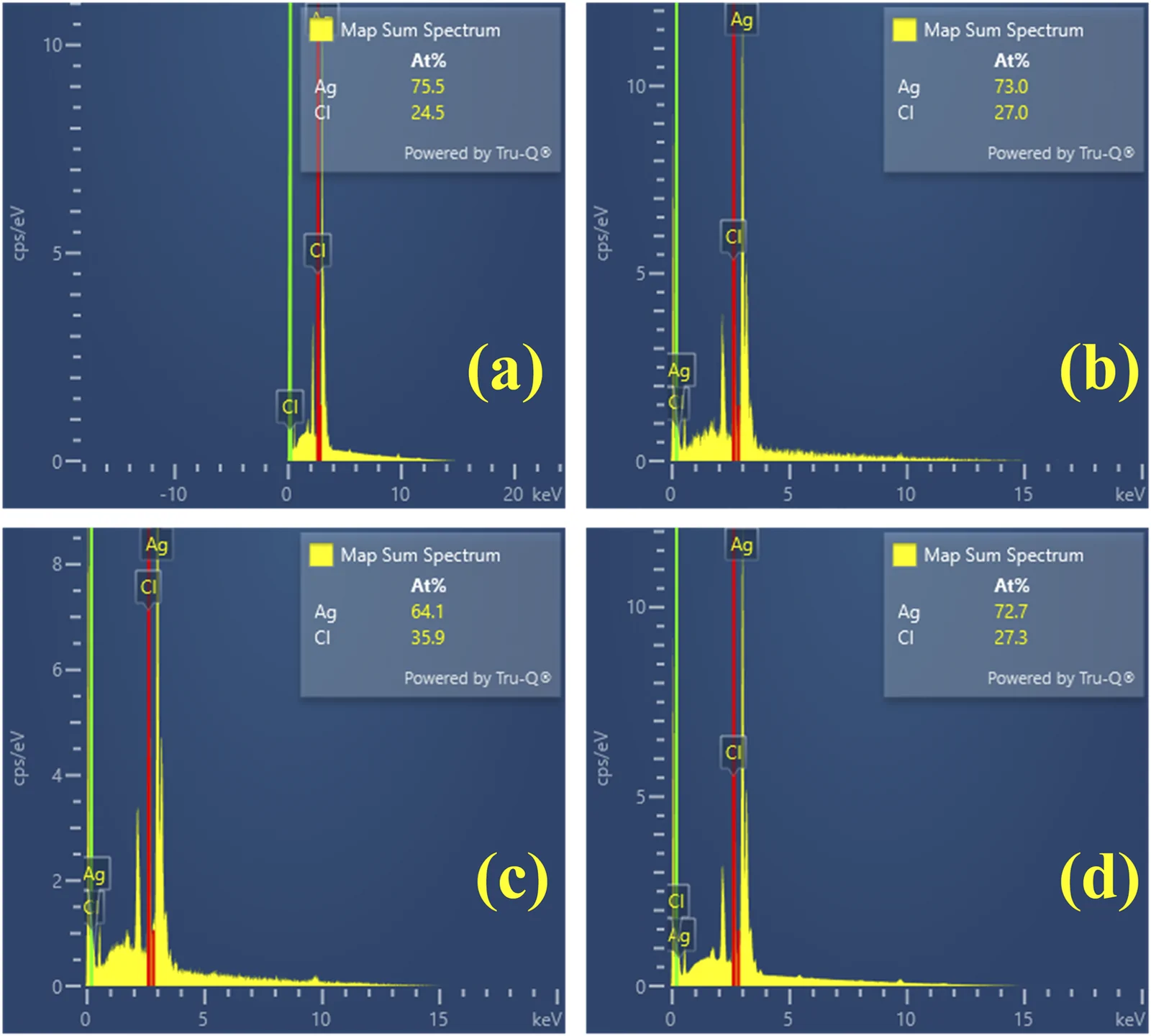
Representative EDS spectra and elemental maps of baking paper substrate after 0.5 wt% NaClO chlorination for **(a)** 120 s, **(b)** 180 s, **(c)** 240 s, and **(d)** 300 s. Data acquired from at least three randomly selected regions per sample. Representative data are from multiple independent measurements.

**FIGURE 7:**
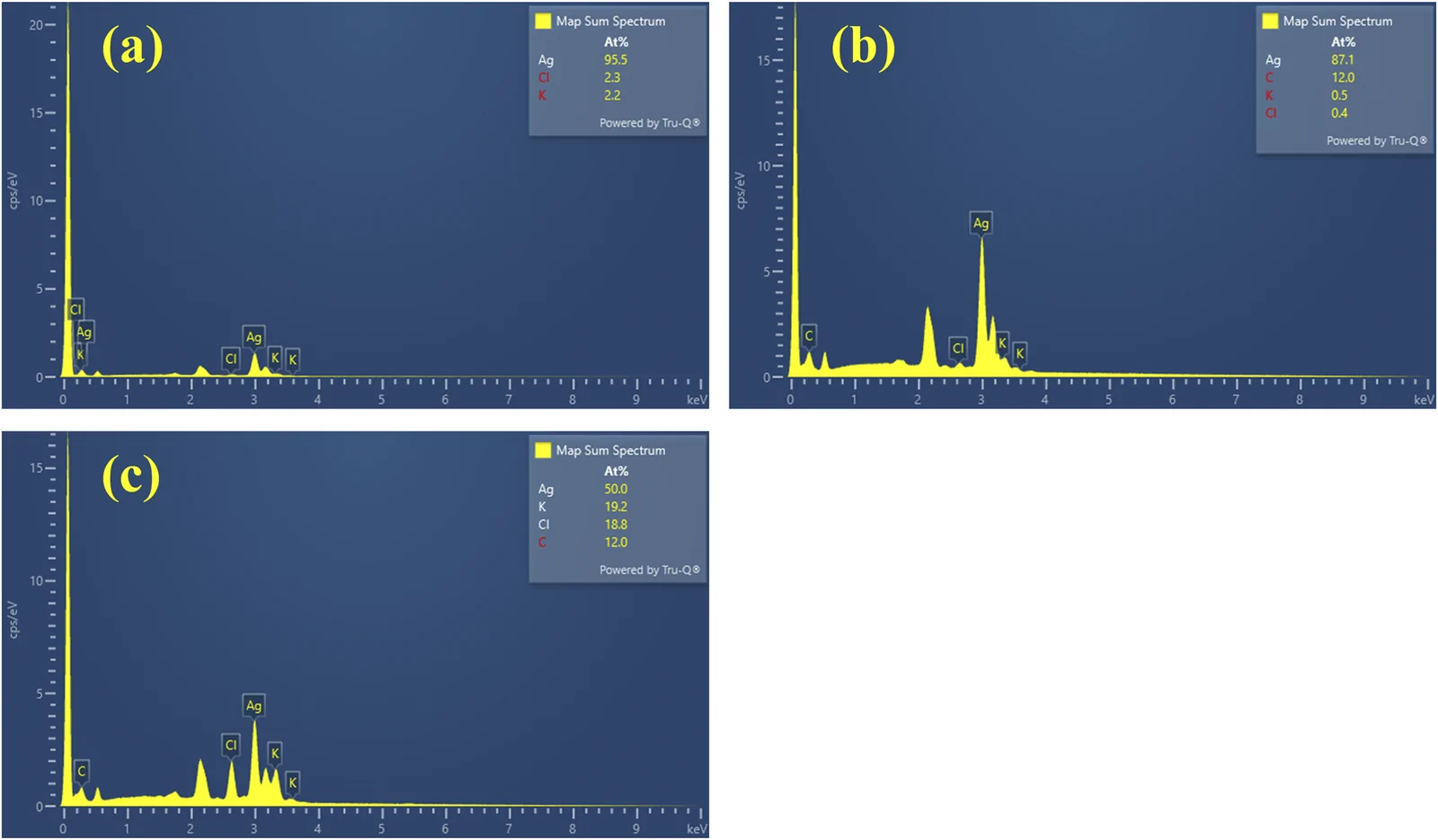
Representative EDS spectra and elemental maps of baking paper substrate after 14 wt% NaClO chlorination for **(a)** 60 s, **(b)** 80 s, and **(c)** 100 s. Representative data are from multiple independent measurements.

EDS point scanning and elemental mapping quantified AgCl conversion uniformity across substrates and chlorination durations. For the baking paper substrate shown in Figures 6, 7, EDS point analysis results indicated that as the chlorination time was extended from 30 s to 240 s, the characteristic X-ray count intensity of Cl element in the characterized area increased significantly, and its atomic ratio to Ag element increased gradually and eventually stabilized. This is consistent with the progressive conversion of Ag to AgCl as reaction time increased.

Meanwhile, the elemental mapping images of Cl element showed that the Cl signal intensity was uniform throughout the observed area, with no obvious element-depleted or aggregated areas. This chemical uniformity is consistent with the fine, dense, and continuous AgCl film morphology observed by SEM, which together illustrate that on the flexible porous substrate, low-concentration NaClO solution combined with appropriate reaction time can achieve uniform and sufficient chlorination, thereby providing a stable Ag/AgCl interface for the electrode.

**FIGURE 8:**
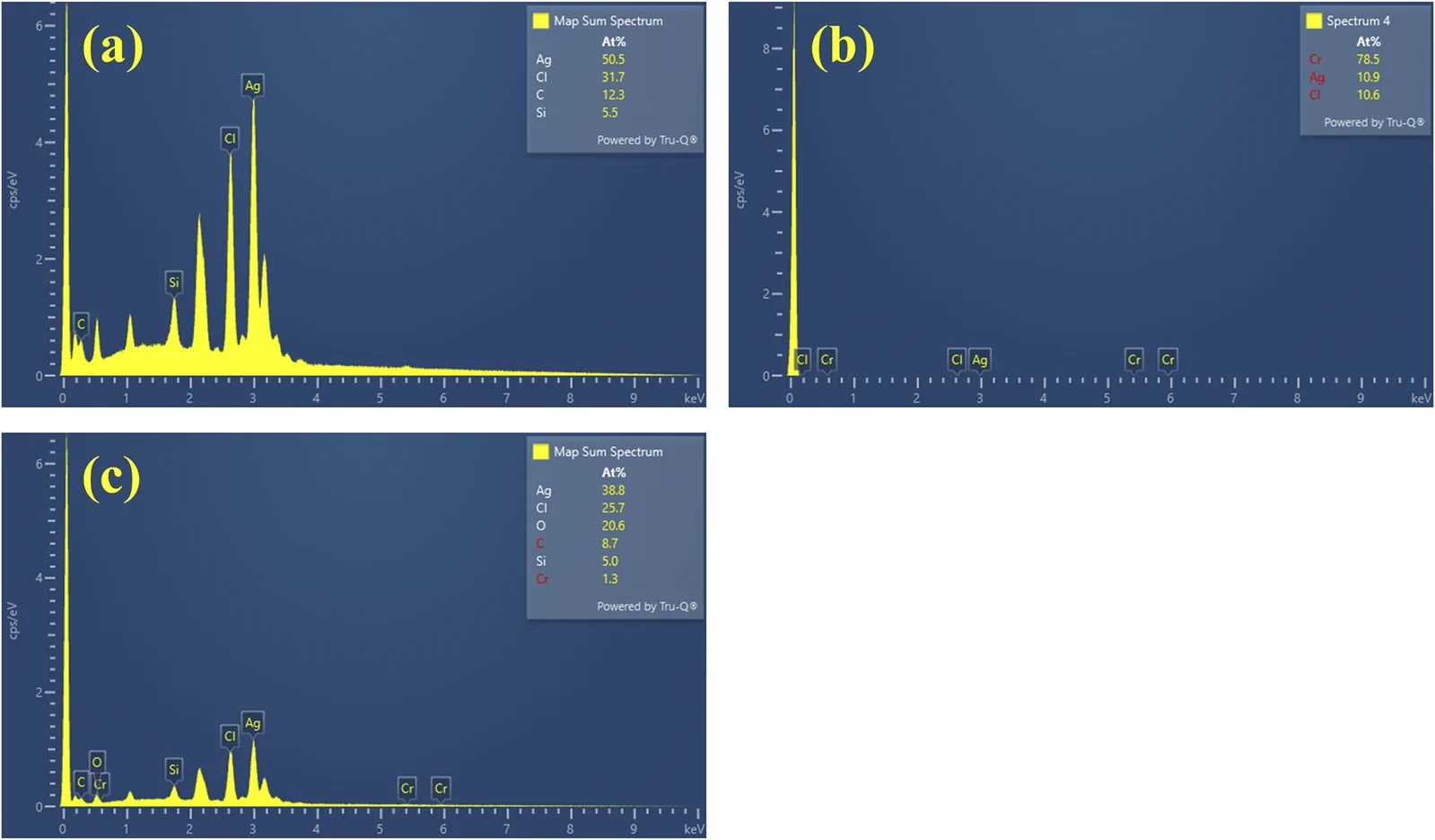
Representative EDS spectra and elemental maps of smooth glass substrate after 0.5 wt% NaClO chlorination for **(a)** 90 s, **(b)** 120 s, and **(c)** 150 s. Representative data are from multiple independent measurements.

For the smooth glass substrate, EDS analysis results further revealed the sensitivity of its AgCl formation process to reaction time and its inherent inhomogeneity, as shown in Figure 8. Under the condition of 0.5 wt% NaClO, point analysis showed that the Cl/Ag atomic ratio increased rapidly with prolonged chlorination time. However, when the chlorination time was extended beyond its optimal window (90 s), the elemental mapping images of Cl exhibited significant inhomogeneity. Abnormal Cl enrichment was observed in local areas, while strong Ag signals were still detected in other regions.

The Cl distribution inhomogeneity observed in EDS mapping (Figure 8) correlated directly with the microcracks and delamination seen in SEM (Section 4.2), resulting in Cl-depleted areas and exposed Ag/Cr signals.

**FIGURE 9:**
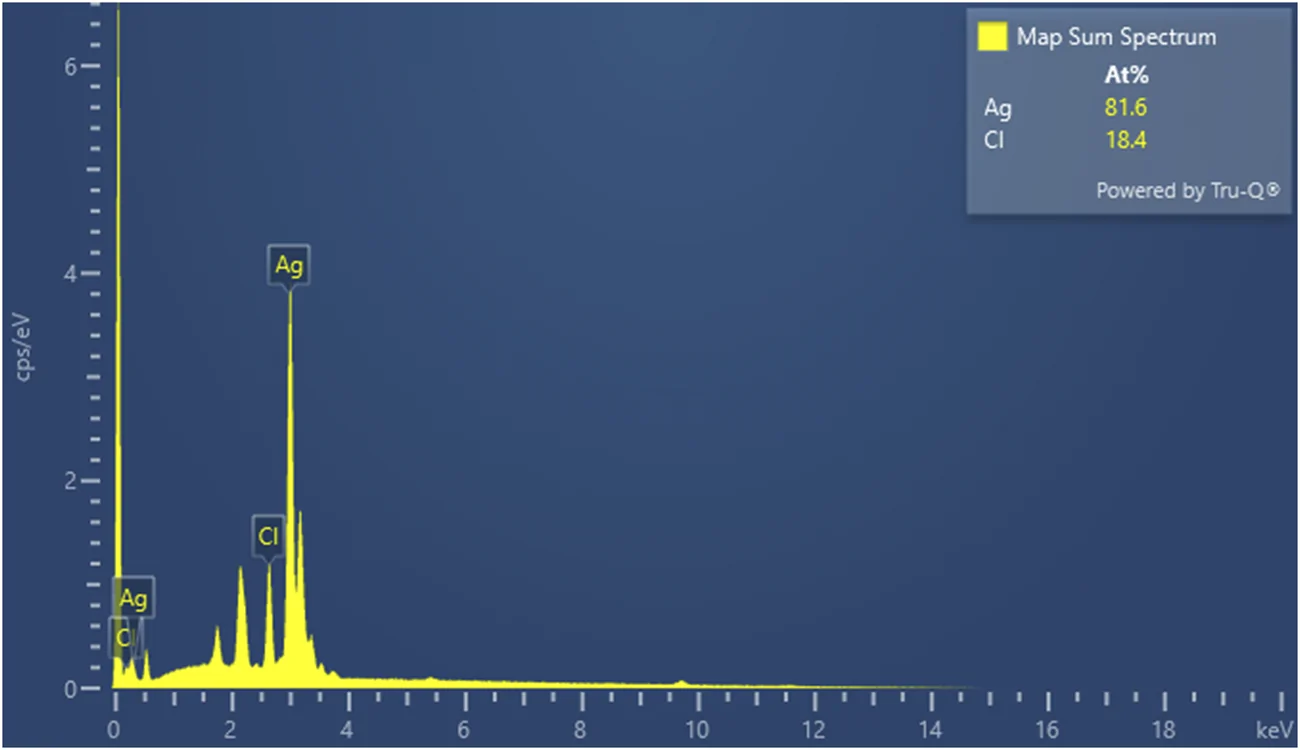
Representative EDS spectrum and elemental map of roughened glass substrate after 210 s of 0.5 wt% NaClO chlorination. Representative data are from multiple independent measurements.

For the roughened glass substrate, its artificial microtextured surface exhibited distinct advantages in the EDS analysis, as shown in Figure 9. The elemental mapping images showed that the distribution of Cl element throughout the observed area maintained good uniformity, without the obvious coexistence of enriched and depleted areas as observed on the smooth glass substrate. Meanwhile, the quantitative point analysis results indicated that the time-dependent curve of the Cl/Ag atomic ratio was flatter and less fluctuating compared with that of the smooth substrate.

Similarly, only one EDS data point at 210 s is shown because it represents the fully reacted, uniform, and stable state of the AgCl layer. The evolution trend of Cl/Ag ratio is analogous to that of smooth glass, so additional time points do not enhance the interpretation.

At an acceleration voltage of 10 kV used in this study, the electron–sample interaction volume for EDX detection is approximately 1–3 μm, which fully covers the thickness of the Ag/AgCl layer and ensures that the collected signals mainly reflect the surface composition of the functional film rather than the underlying substrate. However, tiny microcracks or locally thinned areas of AgCl films allow incident electrons to penetrate through the coating and excite Si signals from glass substrate, which accounts for the detectable silicon peak in partial EDS spectra. All EDX spectra were obtained from at least three randomly selected regions on each sample to avoid local inhomogeneity, and the presented data represent typical and statistically repeatable results. In particular, the relatively weak Cl signal observed in Figure 7b is physically reasonable and statistically valid. At the initial stage of chlorination, only sparse AgCl nuclei are formed, leading to low Cl coverage and low signal intensity. This weak signal does not indicate invalid data but objectively reflects the incomplete nucleation state at the early reaction stage. The consistency of the time-dependent variation in Cl/Ag ratio further verifies the reliability of the entire EDX dataset.

The improved uniformity and stability of the chemical composition on roughened glass, compared to smooth glass, suggest that the rough surface may enhance film adhesion through increased interfacial contact area and provide a more homogeneous reaction environment for chlorination. This interpretation is consistent with the SEM observations.

The color scales shown in the EDS elemental mapping images are generated automatically by the instrument software for visualization purposes. Any negative values appearing in the color bars do not correspond to negative elemental concentrations or negative X-ray counts, but arise solely from image normalization and contrast enhancement procedures. Therefore, they do not affect the interpretation of elemental distribution or the conclusions of this study.

### 4.4 OCP stability analysis

**FIGURE 10:**
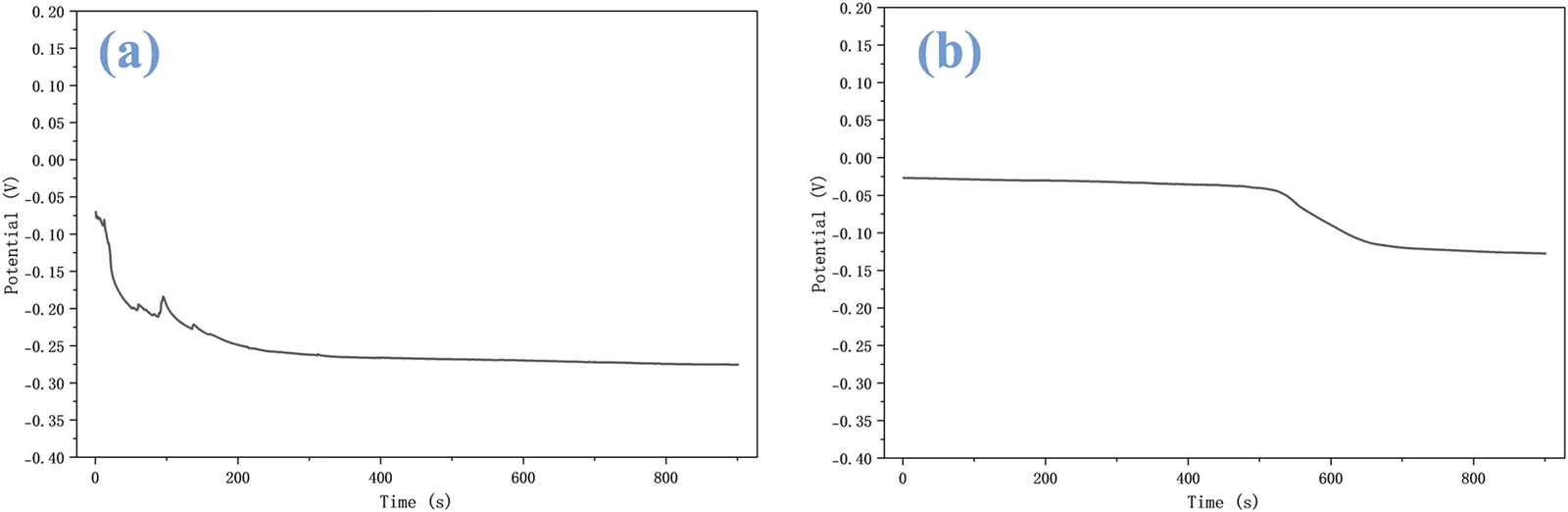
Representative OCP curves (vs. commercial Ag/AgCl (KCl) RE) of baking paper-based PRE in 3.5 M KCl electrolyte, chlorinated with 0.5 wt% NaClO for **(a)** 240 s and **(b)** 300 s. Representative data are from multiple independent measurements.

**FIGURE 11:**
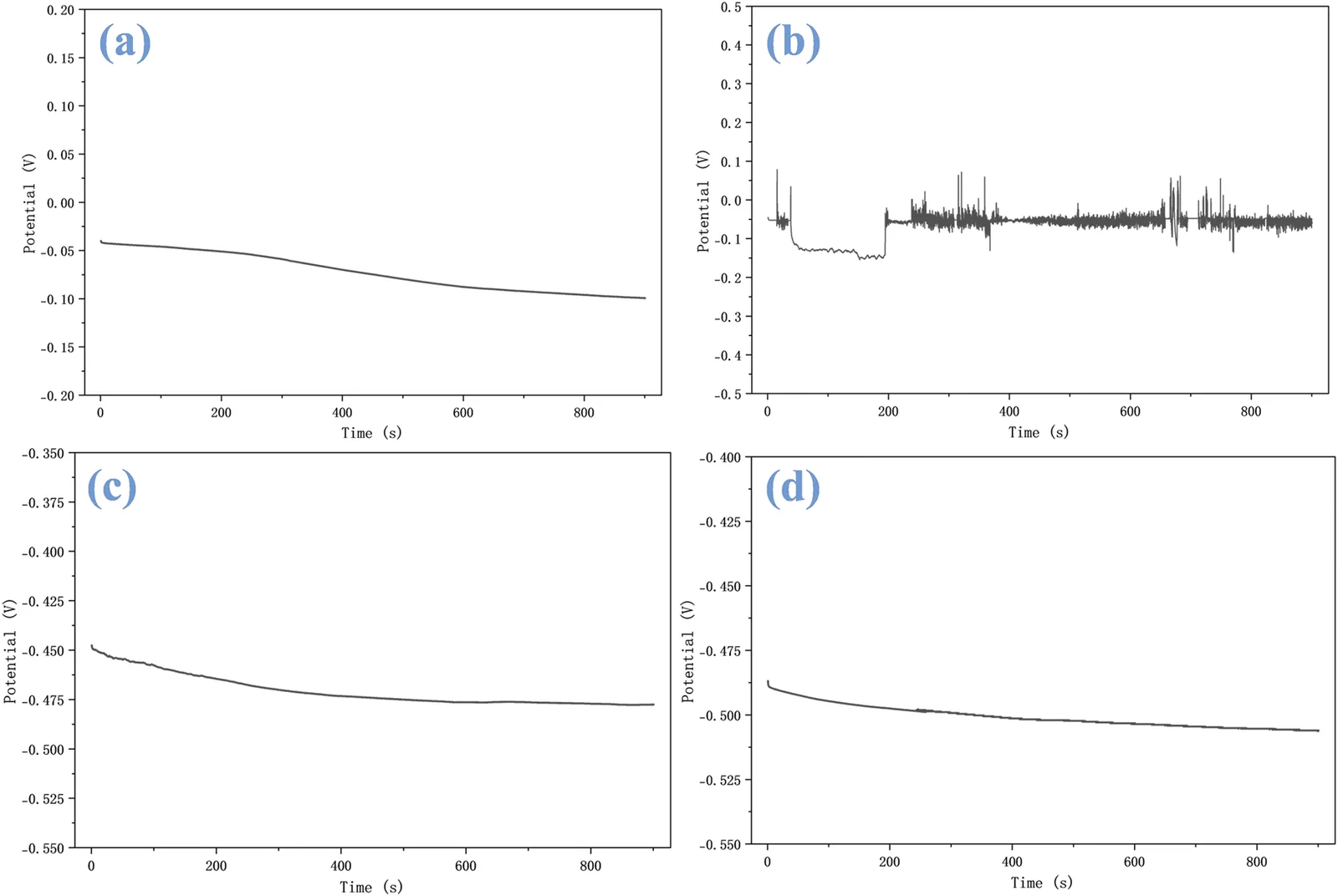
Representative OCP curves (vs. commercial Ag/AgCl (KCl) RE) of baking paper-based PRE in 3.5 M KCl electrolyte, chlorinated with 14 wt% NaClO for **(a)** 40 s, **(b)** 60 s, **(c)** 80 s, and **(d)** 100 s. Representative data are from multiple independent measurements.

The baking paper substrate samples exhibited the optimal stability. Under the conditions of 0.5 wt% NaClO and 240 s chlorination, their OCP curves showed minor fluctuations with a low drift rate of 0.12 ± 0.03 mV/h, and the potential noise was within ±(1.8 ± 0.5) mV, indicating the formation of an electrochemically stable AgCl layer, representative OCP curves as shown in Figures 10, 11.

**FIGURE 12:**
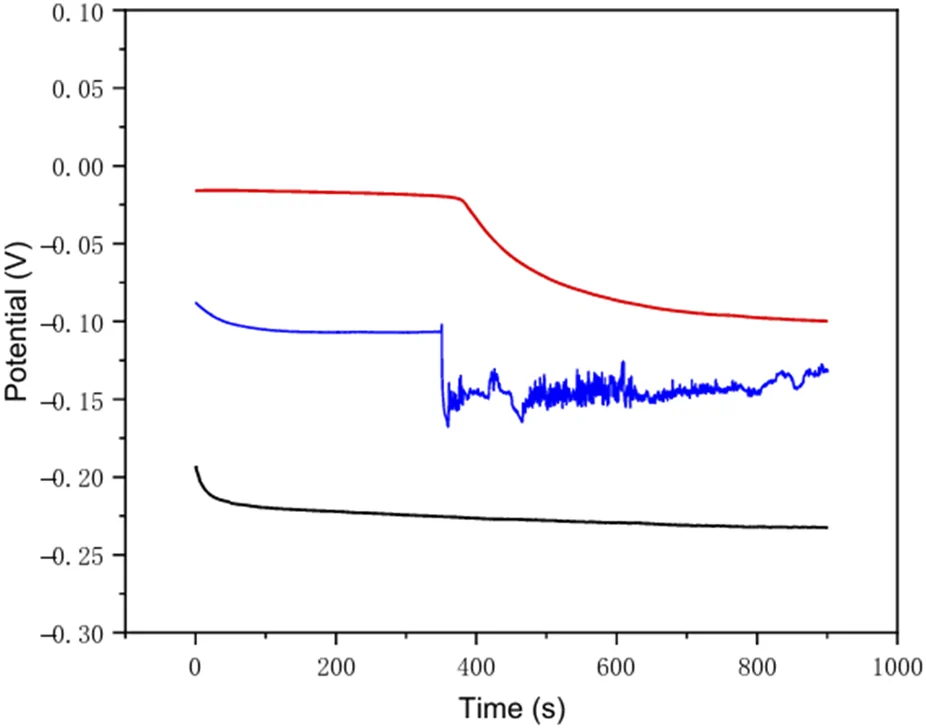
Representative OCP curves (vs. commercial Ag/AgCl (KCl) RE) of smooth glass-based PRE in 3.5 M KCl electrolyte, chlorinated with 0.5 wt% NaClO for 90 s (black), 120 s (red), and 150 s (blue). Representative data are from multiple independent measurements.

In contrast, the smooth glass substrate shown in Figure 12 exhibited extreme sensitivity to chlorination time. The 90 s sample shows the smallest potential drift rate of 0.35 ± 0.08 mV/h, indicating the optimal stable state. The 120 s sample exhibits the largest potential drift rate of 3.21 ± 0.47 mV/h and obvious fluctuations of 9.8 ± 2.3 mV, caused by severe film delamination and structural failure. The 150 s sample shows moderate drift 1.85 ± 0.32 mV/h due to further film degradation.

**FIGURE 13:**
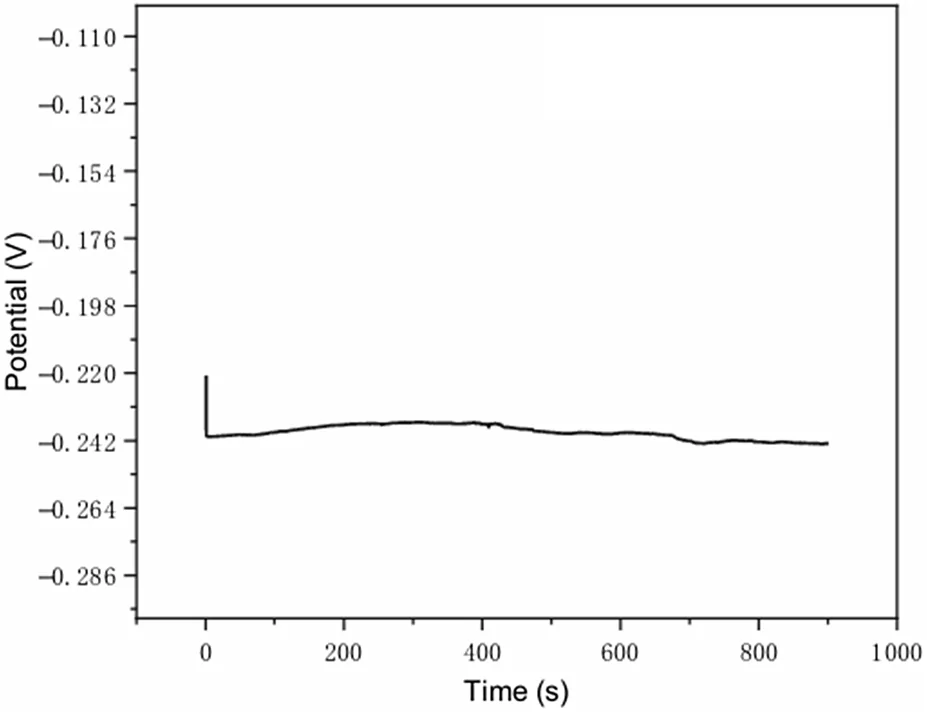
Representative OCP curve (vs. commercial Ag/AgCl (KCl) RE) of roughened glass-based PRE in 3.5 M KCl electrolyte, chlorinated with 0.5 wt% NaClO for 210 s. Representative data are from multiple independent measurements.

The OCP performance of the roughened glass substrate fell between those of the other two substrates. As seen in Figure 13, under low-concentration NaClO conditions, its potential drift rate of 0.22 ± 0.05 mV/h was lower than that of the smooth glass substrate 0.35 ± 0.08 mV/h, indicating a positive effect of the rough structure on stability. However, it still exhibited a certain degree of fluctuation under high-concentration conditions, which corresponded to the increased particle size and local stress-induced delamination observed via SEM. Overall, the OCP results were consistent with the structural rules revealed by SEM and EDS, namely, that a dense, uniform and strongly adherent AgCl film is the key to achieving stable potential output.

Notably, obvious differences in equilibrium OCP values are observed among PRE on different substrates under optimal conditions. The baking paper-based PRE shows a relatively positive and stable potential, while the smooth glass PRE exhibits a clear negative shift, and the roughened glass PRE lies in between. These potential differences originate from the AgCl layer quality and interfacial state: the flexible, porous baking paper facilitates a complete Ag/AgCl/Cl^−^ equilibrium; the smooth glass suffers from weak adhesion and incomplete interface; the roughened glass provides better anchoring and thus a more reliable interface. Such voltage differences reflect the intrinsic interfacial stability dominated by substrate properties.

### 4.5 CV electrochemical behavior analysis

**FIGURE 14:**
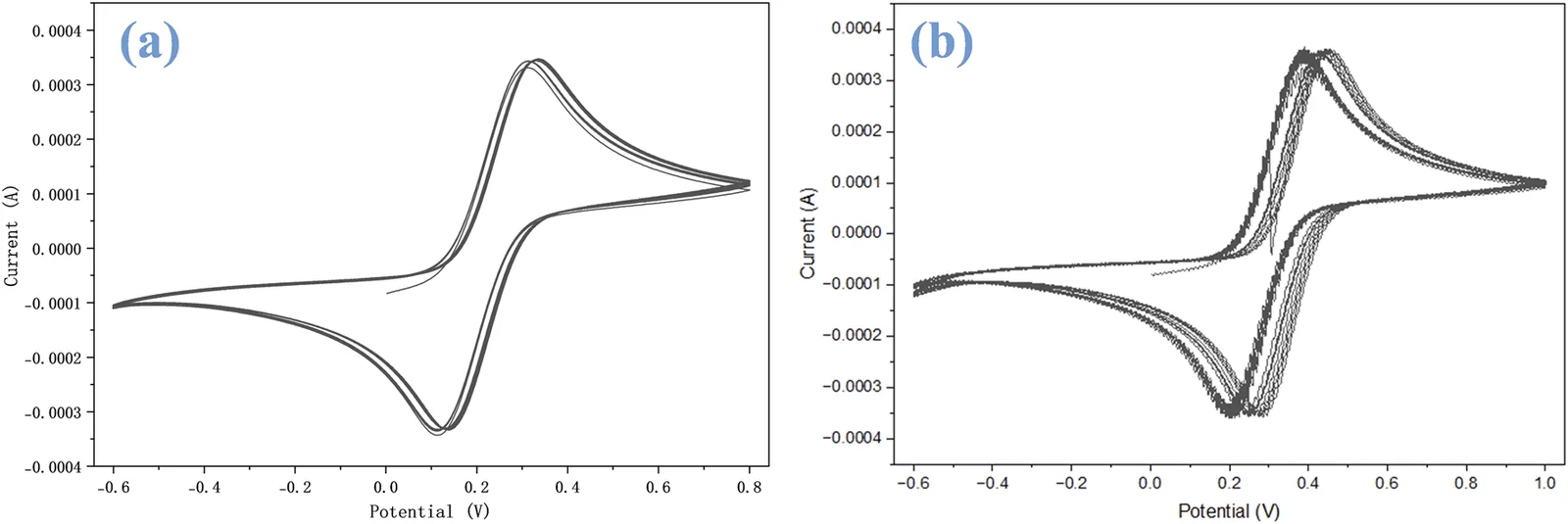
Representative CV curves (15 cycles, 50 mV/s, in 3.5 M KCl + 1 mM K_3_ [Fe(CN)_6_]/K_4_ [Fe(CN)_6_]) of baking paper-based PRE chlorinated with 0.5 wt% NaClO for **(a)** 240 s and **(b)** 300 s. Representative data are from multiple independent measurements.

**FIGURE 15:**
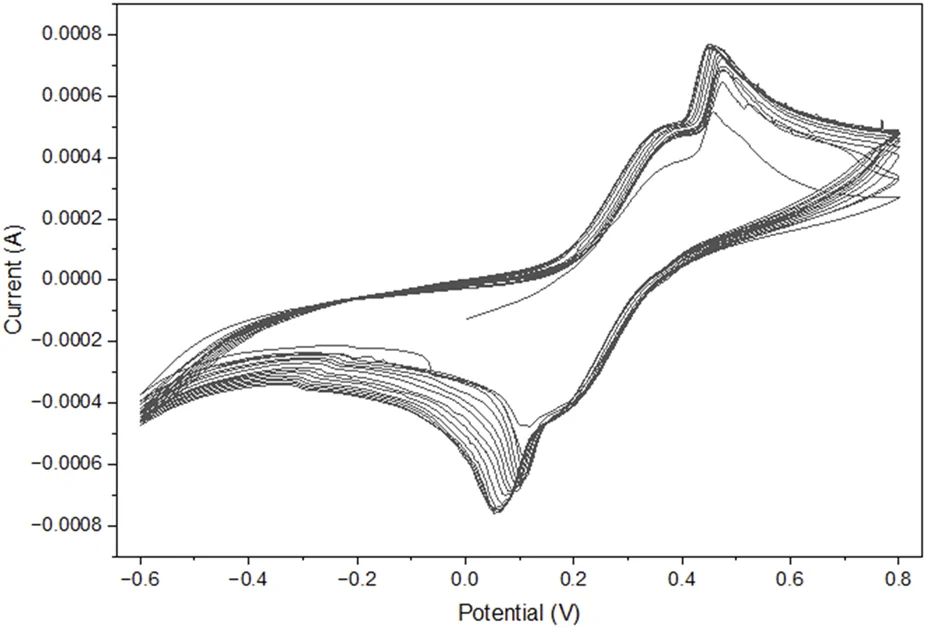
Representative CV curves (15 cycles, 50 mV/s, in 3.5 M KCl + 1 mM K_3_ [Fe(CN)_6_]/K_4_ [Fe(CN)_6_]) of baking paper-based PRE chlorinated with 14 wt% NaClO for 40 s. Representative data are from multiple independent measurements.

CV measurements in ferricyanide redox electrolyte compared dynamic cycling stability of PREs on three substrates, as shown in Figures 14, 15. The baking paper-based PRE (0.5 wt%, 240 s) exhibited an anodic peak current (Ipa) of 12.4 ± 0.6 μA with a peak-to-peak separation (ΔEp) of 78 ± 3 mV. The roughened glass electrode (0.5 wt%, 210 s) delivered highly overlapped cyclic curves with Ipa of 11.5 ± 0.7 μA and ΔEp of 82 ± 4 mV, presenting the best cycling stability among glass substrates. By contrast, smooth glass (0.5 wt%, 90 s) yields broad, asymmetric peaks with Ipa of 9.8 ± 0.9 μA, ΔEp of 95 ± 6 mV, and an anodic peak shift of 12.6 ± 2.4 mV over 15 cycles, resulting from discontinuous, easily detached AgCl film restricting stable interfacial charge transfer.

**FIGURE 16:**
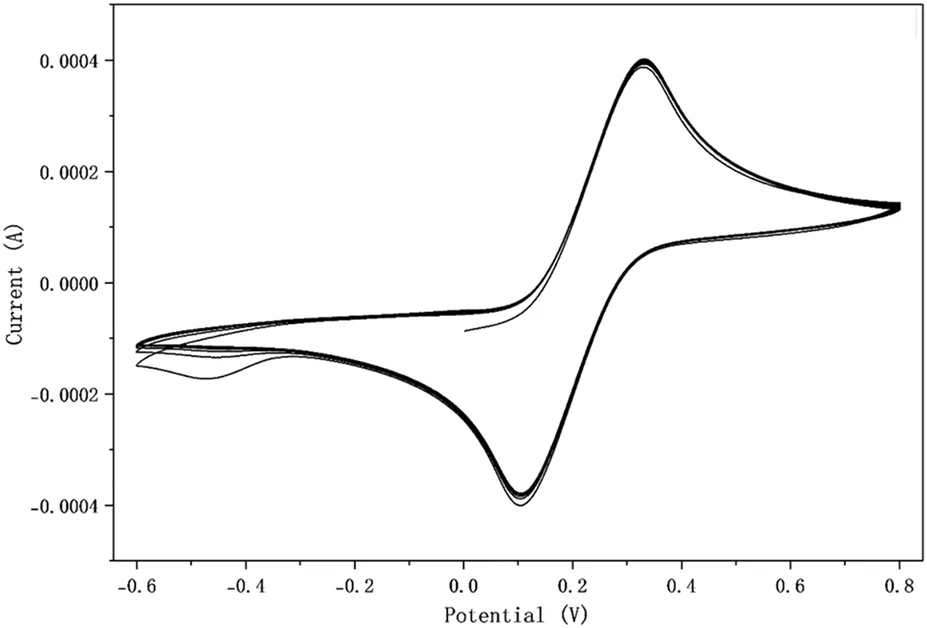
Representative CV curves (15 cycles, 50 mV/s, in 3.5 M KCl + 1 mM K_3_ [Fe(CN)_6_]/K_4_ [Fe(CN)_6_]) of smooth glass-based PRE chlorinated with 0.5 wt% NaClO for 90 s. Representative data are from multiple independent measurements.

**FIGURE 17:**
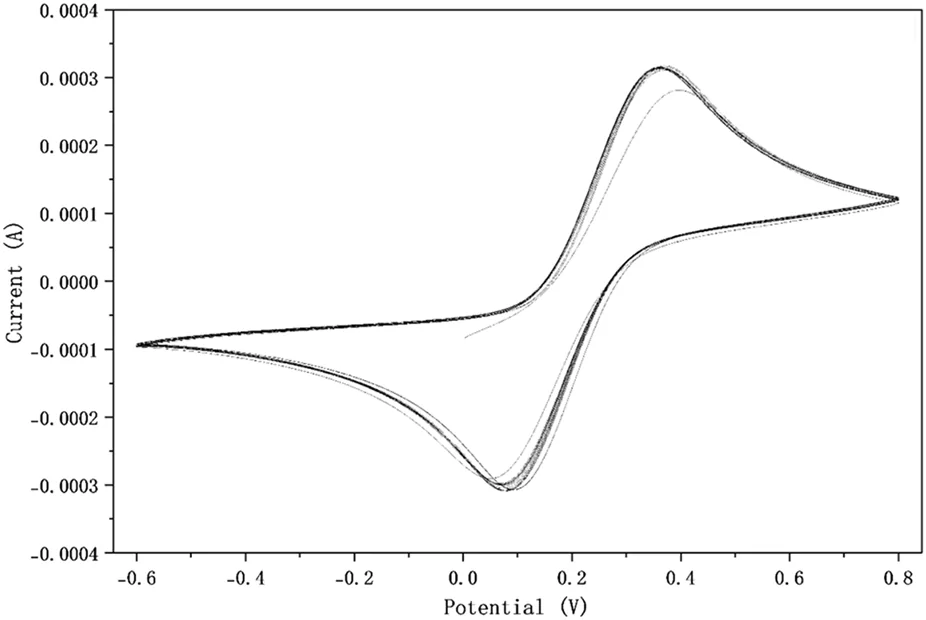
Representative CV curves (15 cycles, 50 mV/s, in 3.5 M KCl + 1 mM K_3_ [Fe(CN)_6_]/K_4_ [Fe(CN)_6_]) of roughened glass-based PRE chlorinated with 0.5 wt% NaClO for 210 s. Representative data are from multiple independent measurements.

Representative CV curves are presented in Figures 14–17. Qualitatively consistent results were obtained from replicate electrodes, confirming the reproducibility of the observed trends. The samples based on the smooth glass substrate exhibited significant apparent peak potential drift. Since the potential axis in CV measurements is defined relative to the reference electrode, the shifts in the redox peak positions shown in Figure 16 reflect the potential instability of the PRE themselves. When the chlorination is insufficient or the Ag/AgCl interface is damaged due to film delamination, the reference electrode cannot establish a stable thermodynamic equilibrium, resulting in potential fluctuations, which are further manifested as random shifts in peak potentials or baseline oscillations in the CV curves. Specifically, the smooth glass substrate samples showed more pronounced peak position shifts.

As seen in Figure 17, the CV performance of the roughened glass substrate outperformed that of the smooth glass substrate but was slightly inferior to that of the baking paper substrate. Its anodic peak potential shift of 4.8 ± 1.1 mV over 15 cycles was significantly lower than that of smooth glass (12.6 ± 2.4 mV), and its peak positions only underwent slight adjustments during the first few scans and then stabilized, indicating that the rough structure can enhance film adhesion to a certain extent, thereby maintaining high consistency in potential output during repeated scans.

### 4.6 Comparison with reported Ag/AgCl pseudo-reference electrodes

To contextualize the performance of the PRE fabricated in this work, Table 2 compares the present electrodes with three representative Ag/AgCl reference electrodes from recent literature, selected to span different fabrication methods (inkjet printing, thermal evaporation, e-beam evaporation) and substrate types, including gold-plated copper (PCB), flexible acetate, and polyimide substrates with a polydimethylsiloxane (PDMS) salt reservoir composite.

**TABLE 2:** Comparison of Ag/AgCl pseudo-reference electrodes from recent literature.

References	Substrate	Fabrication method	Key feature
Papamatthaiou et al. (2020)	PCB	Inkjet printing of Ag nanoparticle ink + chemical sintering	No additional protective coating; 0.04 mV/h drift under continuous flow (24 h)
Torres-Gonzalez et al. (2022)	Flexible acetate	Thermal evaporation of Ag + FeCl_3_ chlorination	Tailorable for planar devices; PDMS salt reservoir composite
Lim et al. (2020)	Polyimide	E-beam evaporation + cyclic electrochemical chlorination	Nafion protective layer; enhanced interfacial adhesion via vacuum annealing; 0.09 mV/h drift for 18 days
This work	Baking paper/Smooth glass/Roughened glass	Magnetron sputtering (Cr/Ag)	No protective coating; systematic comparison of substrate roughness; < 2–5 mV drift (15 min OCP)

Among these, Papamatthaiou et al. (2020) and Torres-Gonzalez et al. (2022) represent protective-layer-free designs like our approach, while Lim et al. (2020) achieved enhanced long-term stability through Nafion coating and interfacial adhesion engineering. The present work contributes the first systematic comparison of substrate roughness effects on PRE, performance under identical chlorination conditions, achieving competitive short-term stability without additional protective coatings.

## 5 Study limitations

Although this work explores the interactions among substrate roughness, chlorination parameters and the performance of Ag/AgCl quasi-reference electrodes, we recognize several limitations in the adopted experimental approaches, which can guide future research and practical applications.

First, we analyze substrate surface roughness qualitatively relying on SEM observations and established preparation procedures, such as sequential grinding and polishing for glass substrates, rather than performing quantitative measurements using atomic force microscopy (AFM) or optical profilometry. Without quantitative roughness indicators including arithmetic mean roughness, root-mean-square roughness and maximum peak-to-valley height, it remains challenging to establish precise quantitative correlations between specific surface features and the observed AgCl nucleation density, grain growth kinetics or interfacial adhesion strength. We believe that incorporating AFM characterization in follow-up work will help develop more rigorous models describing the relationship between microstructure and electrochemical performance.

Second, the evaluation of electrochemical stability is mainly based on short-term OCP drift tests and CV cycling. We have not conducted long-term stability tests, such as continuous multi-day or multi-week open-circuit potential monitoring in different electrolytes. Such long-duration tests are important to confirm the reliability of these quasi-reference electrodes for practical sensing scenarios, where continuous operation over a long period is often required. Besides, electrochemical impedance spectroscopy (EIS) measurements are not included in this study. Electrochemical impedance spectroscopy can offer valuable supplementary information about interfacial charge transfer resistance, thin-film porosity and the evolution of AgCl layer integrity over time. Combining electrochemical impedance spectroscopy with long-term aging tests will deepen our understanding of electrode durability and failure mechanisms.

Third, in the SEM and energy-dispersive X-ray spectroscopy analysis, morphological evolution of AgCl on rough glass substrates is only presented at a single optimized time point, without full time-series results for this substrate. While the overall growth trend on rough glass is expected to be qualitatively similar to that on smooth glass, the lack of complete time-series data covering nucleation, growth and degradation stages prevents fully consistent comparative analysis of chlorination kinetics on the three substrates under identical experimental conditions. This constraint partially limits our interpretation regarding how moderate surface roughness affects the reaction process.

## 6 Conclusion

This work investigates AgCl microstructure and electrochemical behaviors of solid-state Ag/AgCl pseudo-reference electrodes on baking paper, smooth glass and roughened glass with different NaClO treatments. From SEM, EDS, OCP and CV results under current test conditions, substrate roughness, film adhesion and chlorination parameters seem to jointly influence AgCl morphology and electrode potential performance.

In 0.5 wt% NaClO, favorable intact AgCl films form at 240 s on baking paper, 210 s on roughened glass and 90 s on smooth glass. Baking paper’s porous fibrous structure favors AgCl nucleation and stress relief, while rigid smooth glass lacks surface anchoring sites and easily suffers film cracking after over-chlorination. Polished glass gains enhanced AgCl adhesion but cannot mimic baking paper’s flexible fiber network; baking paper was selected for its uniform texture and oil resistance suitable for disposable sensors.

High-concentration 14 wt% NaClO causes coarse AgCl grains and poor film integrity, especially on smooth glass. Tested electrodes roughly follow the stability order: baking paper > roughened glass > smooth glass, and optimized samples can roughly differentiate solutions of different chloride levels under stirring.

Based on present experimental data, pairing moderately rough or fibrous substrates with optimized mild chlorination is a feasible option to prepare low-cost PRE with improved stability for disposable sensors. This study provides basic experimental references for miniaturized electrochemical sensors, and future work will explore modified substrates and practical sample testing.

## Data Availability

The original contributions presented in the study are included in the article/supplementary material, further inquiries can be directed to the corresponding author.
